# Dutch Norms for the Eyberg Child Behavior Inventory: Comparisons with other Western Countries

**DOI:** 10.1007/s10862-017-9639-1

**Published:** 2017-12-02

**Authors:** Joyce Weeland, Jolien van Aar, Geertjan Overbeek

**Affiliations:** 1 Forensic Child and Youth Care, Research Institute of Child Development and Education, University of Amsterdam, Amsterdam, The Netherlands; 2 Preventive Youth Care, Research Institute of Child Development and Education, University of Amsterdam, Amsterdam, The Netherlands

**Keywords:** Children, Disruptive behavior, ECBI, Norm scores, Parent-reports

## Abstract

The Eyberg Child Behavior Inventory (ECBI) is one of the most widely used and well-validated parent rating scales for children’s disruptive behavior. This screening instrument is a short, targetted and easy to implement inventory with good psychometric properties and is normed for different countries, among which the United States, Spain, Sweden and Norway. The ECBI has been successfully used for research and clinical purposes, in several countries including The Netherlands. To date, Dutch studies have relied on Scandinavian or US norm scores. However, this may be problematic because of cross-cultural differences in the degree to which certain behaviors are seen as problematic by parents. The main goal of this paper therefore was to obtain norm scores for The Netherlands among 6462 Dutch children aged 4 to 8 years (*M*_age_ = 6.37 years; *SD* = 1.32; 50.6% boys). In line with previous research, we found small differences on the mean sum scores across children of different ages (intensity scale) and gender (intensity and problem scale). Therefore, Dutch norm scores were provided age- and gender specific. Our results showed that disruptive behavior of children in the most rural areas was reported as occurring less frequently and was seen as less problematic by parents compared to the disruptive behavior of children in less rural areas. Finally, we found that Dutch norm scores on the ECBI were significantly lower than US norm scores, and significantly higher on the intensity scale (but not the problem scale) than Norwegian and Swedish norm scores.

An early onset of disruptive behavior often precedes developmental difficulties in childhood, as well as behavior problems later in life, such as aggression, delinquency, and school problems (Eron and Huesmann 1990; Jokela et al. 2009; Von Stumm et al. 2011). The onset of persistent disruptive behavior can be identified as early as age four (Eron and Huesmann 1990; Newman et al. 1997; Prior et al. 2001). Without intervention, such early behavior problems may develop into persistent patterns of disruptive behavior before the age of eight years (Eron and Huesmann 1990). For example, children who show an increase in aggressive behavior when starting school (in The Netherlands this is the age of 4) have been found to show a distinct temperament, and might be specifically at risk for continuing social and scholastic difficulties throughout school (Kingston and Prior 1995). Thus, early screening of disruptive behavior might be especially important to identify which children are in need of intervention. However, screening instruments are only valuable when a child’s individual score can be compared with the average of his or her peer group.

In doing so, it is pivotal to account for age, gender, and country specific differences. Indeed, differences in the prevalence of disruptive behavior have been observed between different ages, between boys and girls, and between different (European) countries (Berg et al. 1997; Bilenberg 1999; Lahey et al. 2000; Lee et al. 2007; Maughan et al. 2004). In general it has been found that disruptive behavior decreases over time during childhood and that boys score higher than girls (e.g., Maughan et al. 2004). However, the development of behavior over time might also differ between boys and girls. For example, it has been previously found that the prevalence of physical aggression decreases between the ages of 5 and 11 years for girls, but not for boys (Lee et al. 2007). Also, it has been argued that cross-national variation in the prevalence of disruptive behavior –specifically behavior related to ADHD– might be driven by cultural differences (see for a discussion on this matter Timimi and Taylor 2004). The present study therefore focuses on the development of –gender and age-specific– norm scores of the Dutch Eyberg Child Behavior Inventory (ECBI: Eyberg and Pincus 1999) for children aged 4–8 years in The Netherlands.

The ECBI is one of the most widely used and well-validated parent rating scales for disruptive behavior in children 2 to 18 years of age. This screening instrument is very targeted, short (36 items), as well as easy to implement administer, score, and interpret. It therefore has some important advantages over more comprehensive and/or general screening instruments (e.g., the Child Behavior Checklist (Achenbach and Rescorla 2001) or the Strengths and Difficulties Questionnaire (Goodman 2001)). The ECBI askes parents about disruptive child behavior such as whining, temper tantrums and refusal to eat certain food. The scale has been mostly used for assessing behavior of school-aged children. Although the scale was developed for a broader age-range, it might be that for older children other behaviors than those assessed in the ECBI become more important indicators of disruptive behavior such as lying or cheating, lacking guilt, having bad friends and swearing (see Villodas et al. 2015).

Previous psychometric studies showed that the ECBI has good psychometric properties (i.e., good internal consistency, test-retest reliability and good convergent and divergent validity) for different populations (e.g., clinical and non-clinical, different ethnicities) and across different countries (Axberg et al. 2008; Boggs et al. 2010; Burns and Patterson 2010), including The Netherlands (Abrahamse et al. 2015). The ECBI also has been shown to have both discriminative and predictive validity, by indicating children at risk and predicting the further development of disruptive behavior (Abrahamse et al. 2015; Rich and Eyberg 2001). For example, in a recent Dutch study with an at risk sample the intensity scale was found to have good internal consistency (α > .84 over all measurements) and able to measure changes in disruptive behavior after intervention (Weeland et al. 2017). Similar findings have been reported for Dutch samples of incarcerated mothers (Menting et al. 2014), families of low socioeconomic background and ethnic minorities (Leijten et al. 2017). In The Netherlands, the ECBI has been widely used to assess effectiveness of parenting programs targeting disruptive behavior such as Triple P (e.g., Spijkers et al. 2013), Parent–Child Interaction Therapy (e.g., Abrahamse et al. 2016), and The Incredible Years (e.g., Leijten et al. 2017; Posthumus et al. 2012; Weeland et al. 2017).

In terms of construct validity, although developed as a unidimensional instrument, recently it has been suggested that the inventory measures three distinct clusters of behavior, namely 1) oppositional defiant behavior towards adults, 2) inattentive behavior, and 3) conduct problem behavior (Axberg et al. 2008; Weis et al. 2005). This three factor structure was indeed replicated in several studies (e.g., Burns and Patterson 2010), but not found in others (e.g., Butler 2013; Gross et al. 2007, including in a Dutch sample by Abrahamse et al. 2015). For example, Gross et al. (2007) found more support for the validity of the ECBI as a one-dimensional measure for child behavioral problems. It has been suggested that these factors should therefore not be used for screening or assessing treatment outcome research. We therefore treat the ECBI as a unidimensional instrument.

To date, Dutch studies have relied on United State (Burns & Patterson 2001; Robinson et al. 1980), Norwegian (Reedtz et al. 2008) or Swedish norm scores (Axberg et al. 2008). This is problematic, because there are important cross-cultural differences in the degree to which certain behaviors are seen as problematic by parents (Berg et al. 1997; Bilenberg 1999). Indeed, using the ECBI, Scandinavian parents rated their children’s behavior significantly lower on intensity and less problematic (Axberg et al. 2008; Reedtz et al. 2008) than American parents (Burns and Patterson 2001; Robinson et al. 1980). Specifically, Swedish parents rated the intensity of their 5-year old children’s disruptive behavior with an average sumscore of 88.2 (Axberg et al. 2008) whereas American parents rated the intensity of this behavior with an average sumscore of 104.8 (Robinson et al. 1980). A previous Dutch study found indications that these lower normative scores, compared to US children, might be true for Dutch children as well (Abrahamse et al. 2015). By using for example -possibly higher- American norms in a Dutch context, researchers and clinicians alike may overlook a subgroup of children at risk. Likewise, by using possibly lower Scandinavian norms Dutch professionals may end up overestimating problem behavior in Dutch children. Therefore, to prevent a possible over- or underclassification of disruptive behavior in Dutch children, we need to identify ECBI norm scores for the Dutch population itself.

The goal of the current study was to provide norms for the ECBI intensity scale and ECBI problem scale as a measure of disruptive behavior in Dutch children aged 4–8 years. This age group was selected because this is a crucial age in the development of stable disruptive behavior patterns (Eron and Huesmann 1990; Newman et al. 1997; Prior et al. 2001). Both urban and rural municipalities (high and low in population density) were selected for participation, because of possible differences in the prevalence of disruptive behavior between these types of areas (Elgar et al. 2003; Farrell et al. 2005; Hope and Bierman 1998). All families from the targeted municipalities who had children in the targeted age group were approached for participation. In this way we aimed to obtain a representative sample of the Dutch population. We expected disruptive behavior to differ across age (i.e., lower ECBI scores for older children) and gender (i.e., lower ECBI scores for girls). Accounting for age and gender differences, our aim was to provide norm scores for boys and girls of all ages within the sample. A second aim of this paper was to explore cross-country validity of the ECBI norms. We therefore placed the Dutch norm scores in the context of available norm scores of other western countries, specifically American, Norwegian, Swedish and Spanish norm scores. In line with the results of previous European studies, we expected Dutch norm scores to be significantly lower than American norm scores. We explored differences in norm scores between The Netherlands and Norway, Sweden, and Spain. Because these countries might be culturally closer related to The Netherlands than the US, we do not expect differences in norm scores between these countries.

## Method

### Procedure

The procedure of data collection for this study occurred in the context of a randomized controlled trial (Chhangur et al. 2012) and was approved by the Institutional Review Board in The Netherlands (METC UMC Utrecht, protocol number 11–320/K). Informed consent was obtained from all individual families included in the study. The community records of two regional health care centers in The Netherlands were used to identify families with children between 4 and 8 years of age. Both rural and urban municipalities were selected as sampling areas. The final sampling area consisted of four Dutch municipalities: one large city (with a mean of 1510 residents per km^2^), one (middle-) large city (2383 residents per km^2^), and two more rural areas (411 and 657 residents per km^2^). These areas also differed in mean income and immigrant population (both above and below Dutch average, see Table 1). All families in the selected areas with one or more children between 4 and 8 years old received an invitation from the health care organization including an information letter about the study aims, an informed consent form and the ECBI by mail (*N* = 26,055 children of 20,048 families). In the invitation it was explained that the health care organization participated in the study but that they would not receive any information about participants. It was also made clear that participating families might be invited to participate in a second part of the study (i.e., the RCT) but that participation in this was not mandatory. Families with multiple children in the targeted age category received a personalized inventory for each targeted child. Families were asked to return the ECBI’s, together with a signed consent form, within one week. In an attempt to boost the response rate, parents filled out the ECBI in complete anonymity. Participating families received a gift card of € 7.50.

**Table 1:** Descriptive statistics of participating municipalities (year 2013)

Area	Population	Residents between 4 and 8 years	Response (%)	Mean year income per person (€)			Immigrant population (%)			ECBI Intensity scale			ECBI Problem scale		
										*N* ^*b*^	*M*	*SD*	*N*	*M*	*SD*
The Netherlands (total)	16,779,575			22,400			21.0								
				general	*M s*ample	range sample	general	*M* sample	range sample						
Almere	193,165	12,269	21.8	21,900	22,330^c^	16,000–54,000	38.0	38.1	8–65	2647	96.72	28.17	2291	4.06^g^	6.02
Amersfoort	148,250	9047	28.0	24,000	24,520^c^	13,000–47,000	23.0	21.6^c^	5–72	2529	95.87	24.84	2297	3.33	4.98
Woerden area	35,304 ^a^	1480 ^a^	35.2	24,313^a^	24,050^c^	21,000–27,000	3.9^a^	7.9^e^	1–11	521	93.20^f^	24.53	449	2.64^f^	4.19
Stichtse Vecht	63,315	3259	22.5	26,200	27,590^d^	21,000–41,000	17.0	10.5^e^	3–20	733	97.10	25.87	645	3.45	5.24

General = mean for general population of that area; Sample = mean for study sample; Source: CBS 2013

^a^Average across 5 villages

^b^For the intensity and problem scales respectively 32 and 780 cases were removed because they had more than 4 missings

^c^Small significant difference between general and sample population (partialη^2^ < .06*; p* < .01, see for interpretation partial η^2:^ Cohen 1969)

^d^Moderate significant difference between general and sample population (partialη^2^ .06–.14*; p* < .01)

^e^Large significant difference between general and sample population (partialη^2^ > .14*; p* < .01)

^f^Significantly lower than scores of the three other areas

^g^Significantly higher than scores of the three other areas

### The Eyberg Child Behavior Inventory (ECBI)

The Eyberg Child Behavior Inventory (ECBI) is a 36-item questionnaire for parents to measure child conduct behavior (Eyberg and Pincus 1999). It consists of two scales, one “intensity scale” and one “problem scale”. Both scales include the same 36 items that state a specific behavior (e.g., ‘*Refuses to do chores when asked’*). On the Intensity scale, parents report the frequency of the specific behavior using a Likert scale from “never happens” (1) to “always happens” (7). On the Problem scale, parents report whether they perceive the specific behavior as a problem (yes or no).

### Reliability

Reliability for the intensity scale (the problem scale is binary) was assessed in terms of internal consistency (i.e., Cronbach’s alpha), and was excellent (Cronbach’s alpha = .93). Item-total correlations ranged from 0.17 (item 36, *Wets the bed*) to .68 (item 9, *Refuses to obey until threatened with punishment*) with a median of .51. The intensity and problem scales were highly and positively correlated (*r* = .60, *p* < .001), which means that parents who perceived children’s disruptive behavior to be more intense, perceived such behavior as more problematic at the same time.

### Sample

A total of 6462 questionnaires of 5470 families of the 26,055 were returned (24.8%). Of those 5470 families, 4518 families had one child, 913 families had two children, 38 families had three children and one family had 4 children in the range of 4–8 years old. This return rate is comparable to that of the 2008 US norm study (28%: Burns and Patterson 2008). These questionnaires came from 5470 families, of which 952 families had two (*N* = 913), three (*N* = 38) or four (*N* = 1) children within the age range of 4–8 years. Based on participants’ postal codes we compared our sample to the specific area population on mean yearly income and percentage immigrants (see Table 1). For three of the four areas the mean yearly income of our sample was higher than the area’s population mean, and for one area this was lower. For two of the four areas the percentage of immigrants was lower than the area’s population percentage, for one area it was higher and for one area no differences were found (see Table 1). Since the differences were mostly small and in both directions, there seem to be no systematic differences between our sample population and the population of the targeted areas. We therefore conclude that our sample is diverse and representative for the targeted areas.

Children for whom the ECBI was completed were on average 6.37 years of age (*SD* = 1.32) and about half of them were boys (50.6%). Controlling for age and gender, there was a very small but significant effect of the area children lived in on both the ECBI Intensity (*F*(3, 6424) = 2.94; *p* = .032, *η* ^*2*^ = .00) and Problem scale (*F*(3, 5678) = 12.62; *p* < .001, *η* ^*2*^ = .01) (the differences in sample size between the intensity and problem scales were explained by relatively more missing values on the problem scale). Specifically, parents from the most rural area reported less frequent problem behavior and perceived these behaviors to be less of a problem compared to parents of the more rural areas (intensity differences ranged from 2.67 to 3.90, *p* < .05, problem differences ranged from 0.70 to 1.42, *p* < .05, Bonferroni adjusted). In addition, parents from the most urban area perceived their children’s behaviors more often as a problem compared to parents from the other area’s (problem differences ranged from 0.62 to 1.43, *p* < .05, Bonferroni adjusted) (see means per area in Table 1). These results show that to provide national norms it is important to use different types of areas.

### Missing Values

Missing data on the ECBI intensity scale ranged between 0.3% and 4.4% across items. For 34 out of 36 items the percentage of missings was less than 1%. However, items 25 (i.e., *Verbally fights with sisters and brothers*) and 27 (i.e., *Physically fights with sisters and brothers*) were left unanswered relatively more often (in 4.4% of the cases), possibly because for children without siblings these items are not applicable. This difference in frequency of missings between these and other items has been reported before (Reedtz et al. 2008). Following the manual by Eyberg and Pincus (1999), cases who left more than four items unanswered on the intensity scale were omitted from norm score analyses (*n* = 32, 0.5%). For cases with four missing items or less missing data was handled using full information maximum likelihood (FIML) estimations. This means that a likelihood function for each individual was estimated based on the variables that are present so that all the available data are used. FIML has been shown to be a very accurate procedure to deal with missingness, specifically compared to listwise deletion or mean imputation (Wothke 2000). The cases who had more than 4 missings on the intensity scale did not significantly differ from the cases with less missings on child age or gender (*p*s > .15), but they did differ on living area (*F* (1, 6459) = 17.60, *p* < .001), it was lowest in the middle large city but highest in the large city. This might be explained by the order in which participants were recruited (participants from the large city were recruited first). The high amount of missing items on the intensity scale in the first cohort might have led to more emphasis on completely filling out the questionnaire by adding a note (i.e., “NOTE: Also indicate in the right hand column whether behavior is currently a problem for you”).

Missing data on the ECBI problem scale ranged between 8.0% and 13.0% across items. Again, a higher percentage of missings (12.4% and 13.0%) was found for the two items about verbally or physically fighting with brothers and sisters. The fact that there were more missing data on the problem scale than on the intensity scale might be explained by the fact that the entire problem scale was often left unanswered. This might indicate that the instructions for this scale were possibly unclear for some parents. Again, cases who left more than four items unanswered were omitted from norm-score analyses (*n* = 780, 12.1%) and other missing values were estimated using FIML procedure. The cases who had more than 4 missings on the problem scale did not significantly differ from the cases with less missings on child age, gender and living area (*p*s > .05). Descriptive statistics per item are presented in Table 2.

**Table 2:** Mean scores per ECBI item

		Intensity		Problem (%)	
		*M*	*SD*		
1.	Dawdles in getting dressed	3.48	1.57	11.80	
2.	Dawdles or lingers at mealtime	3.66	1.67	19.50	
3.	Has poor table manners	2.65	1.29	9.40	
4.	Refuses to eat food presented	2.85	1.46	13.60	
5.	Refuses to do chores when asked	2.88	1.26	10.00	
6.	Slow in getting ready for bed	3.35	1.49	11.80	
7.	Refuses to go to bed on time	2.21	1.37	8.60	
8.	Does not obey house rules on his/her own	2.78	1.24	9.20	
9.	Refuses to obey until threatened with punishment	3.12	1.37	17.50	
10.	Acts defiant when told to do something	3.08	1.27	12.60	
11.	Argues with parents about rules	2.99	1.44	8.50	
12.	Gets angry when doesn’t get his/her own way	3.70	1.36	16.20	
13.	Has temper tantrums	2.57	1.45	13.20	
14.	Sasses adults	2.09	1.18	9.70	
15.	Whines	2.60	1.35	8.30	
16.	Cries easily	2.96	1.48	10.40	
17.	Yells or screams	2.83	1.49	14.90	
18.	Hits parents	1.48	0.93	6.60	
19.	Destroys toys and other objects	1.64	1.05	4.80	
20.	Is careless with toys and other objects	2.04	1.30	5.80	
21.	Steals	1.18	0.59	3.00	
22.	Lies	2.05	1.13	10.30	
23.	Teases or provokes other children	2.23	1.25	6.50	
24.	Verbally fights with friends his/her own age	2.41	1.20	4.70	
25.	Verbally fights with sisters and brothers	3.46	1.51	15.70	
26.	Physically fights with friends his/her own age	1.50	0.89	3.60	
27.	Physically fights with sisters and brothers	2.20	1.43	9.60	
28.	Constantly seeks attention	3.31	1.52	10.90	
29.	Interrupts	3.46	1.44	5.30	
30.	Is easily distracted	3.47	1.62	11.50	
31.	Has short attention span	3.07	1.57	9.00	
32.	Fails to finish tasks or projects	2.66	1.39	7.00	
33.	Has difficulty entertaining him/herself alone	2.71	1.53	9.10	
34.	Has difficulty concentrating on one thing	2.69	1.48	6.50	
35.	Is overactive or restless	2.63	1.64	7.80	
36.	Wets the bed	1.85	1.58	6.10	

## Results

### Means and Percentiles

For the whole sample the mean sum score for the Intensity scale was 95.78 (*SD* = 26.28) and for the Problem scale 3.19 (*SD* = 5.10). Mean sum scores and 75th, 90th, 95th and 98th percentiles are presented in Tables 3 and 4. Behaviors that parents frequently reported (15% of parents or more) as being a problem were: ‘*Gets angry when doesn’t get his/her own way*’ (item 12, *M*_intensity_ = 3.70); ‘*Dawdles or lingers at mealtime’* (item 2, *M*_intensity_ = 3.66), ‘*Refuses to obey until threatened with punishment*’ (item 9, *M*_intensity_ = 3.12); and ‘*Verbally fights with sisters and brothers*’ (item 25, *M*_intensity_ = 3.46).

**Table 3:** Norm Scores and Percentiles for the ECBI intensity Scale

Age	Gender	*N* ^*a*^	Intensity scale		Percentiles				
			*M*	*SD*	75th	90th	95th	98th	
all	Total	6425	96.15	26.38	113.00	131.12	142.99	155.99	
	Boys	3253	99.54	27.05	117.00	136.01	146.99	159.98	
	Girls	3171	92.66	25.21	109.04	124.99	137.02	150.79	
4	Total	1297	99.08	25.12	115.99	132.47	141.01	156.03	
	Boys	662	102.85	25.48	118.94	136.41	146.99	164.21	
	Girls	635	95.16	24.13	112.00	126.71	135.20	145.29	
5	Total	1422	97.98	26.42	114.98	133.52	145.01	155.08	
	Boys	760	100.75	26.94	117.89	137.89	148.00	158.79	
	Girls	662	94.80	25.46	110.99	126.00	137.99	152.48	
6	Total	1335	95.01	25.44	112.00	128.02	141.01	153.00	
	Boys	676	97.51	25.84	114.98	130.00	142.99	154.47	
	Girls	658	92.43	24.79	109.01	124.90	136.06	151.64	
7	Total	1472	94.48	27.00	110.99	131.71	144.00	156.53	
	Boys	720	99.06	28.13	115.99	138.98	149.00	167.59	
	Girls	752	90.09	25.12	105.98	123.95	135.70	148.94	
8	Total	899	93.43	27.85	110.99	132.01	144.00	160.99	
	Boys	435	96.36	28.95	113.00	136.89	149.60	167.29	
	Girls	464	90.68	26.53	108.00	126.00	140.00	155.80	

^a^32 cases were omitted from norm score analyses because they left more than four items on this scale unanswered

**Table 4:** Norm scores and percentiles for the ECBI problem scale

			Problem scale		Percentiles			
Age	Gender	*N* ^*a*^	*M*	*SD*	75th	90th	95th	98th
all	Total	5679^a^	3.59	5.43	5.00	11.02	15.01	20.99
	Boys	2875	3.98	5.77	6.01	12.35	16.39	21.60
	Girls	2803	3.18	5.00	5.00	9.25	14.00	19.01
4	Total	1157	3.55	5.22	5.00	11.02	14.40	18.00
	Boys	588	4.02	5.61	6.01	11.99	15.01	20.99
	Girls	569	3.07	4.74	5.00	9.00	13.00	16.78
5	Total	1265	3.74	5.65	6.01	11.99	15.98	20.99
	Boys	676	4.08	5.91	6.01	12.71	17.14	22.11
	Girls	589	3.35	5.32	5.00	10.01	14.71	19.21
6	Total	1180	3.53	5.44	5.00	11.02	15.75	20.99
	Boys	596	3.60	5.47	5.00	11.76	15.77	21.62
	Girls	583	3.40	5.23	5.00	10.18	15.36	20.99
7	Total	1274	3.50	5.28	5.00	10.01	15.01	20.29
	Boys	630	4.06	5.69	6.01	12.31	16.92	22.13
	Girls	644	2.95	4.78	4.10	9.00	13.75	19.01
8	Total	803	3.65	5.63	5.00	11.99	15.98	21.55
	Boys	385	4.22	6.29	6.01	14.00	19.01	22.75
	Girls	418	3.12	4.89	5.00	9.00	13.00	19.63

^a^780 cases were omitted from norm score analyses because they left more than four items on this scale unanswered

### Comparisons between Age-Groups

Correlation analyses showed that the Intensity scale was related to age (*n* = 6425, *r* = −.08, *p* = < .01), but the Problem scale was not (*n* = 6579, *r* = −.00, *p* = .93). This means that in general parents report slightly less intense problem behavior for older children (but do not report these behaviors as less problematic). Post hoc comparison analyses showed that specifically children aged 4 and 5 years were scored significantly higher than children aged 6, 7, or 8 years (*M*_difference_ ranged from 2.97 to 5.67, *p*s > .02, Bonferroni adjusted). This indicates that in general disruptive behavior decreases after the age of 5. However, parents still might perceive less frequent disruptive behavior in older children as relatively problematic compared to younger children. Parents might thus have different expectations for behavior of older children compared to the behavior of younger children.

### Comparisons between Genders

There were small significant differences between parents’ ratings of boys and girls on the Intensity scale (*F*(1, 6422) = 111.12, *p* < .001, *η* ^*2*^ = .02) and Problem scale (*F*(1, 5676) = 31.21, *p* < .001, *η* ^*2*^ = .01). Parents scored disruptive behavior of boys as more frequently occurring and perceived these behaviors more as a problem than the behavior of girls. Therefore, mean sumscores, as well as 75th, 90th, 95th and 98th percentiles are presented separately for boys and girls per age (Tables 3 and 4). This might indicate that boys show more disruptive behavior than girls or that parents rate behavior differently for sons and daughters.

### Comparisons between Countries

**Fig. 1:**
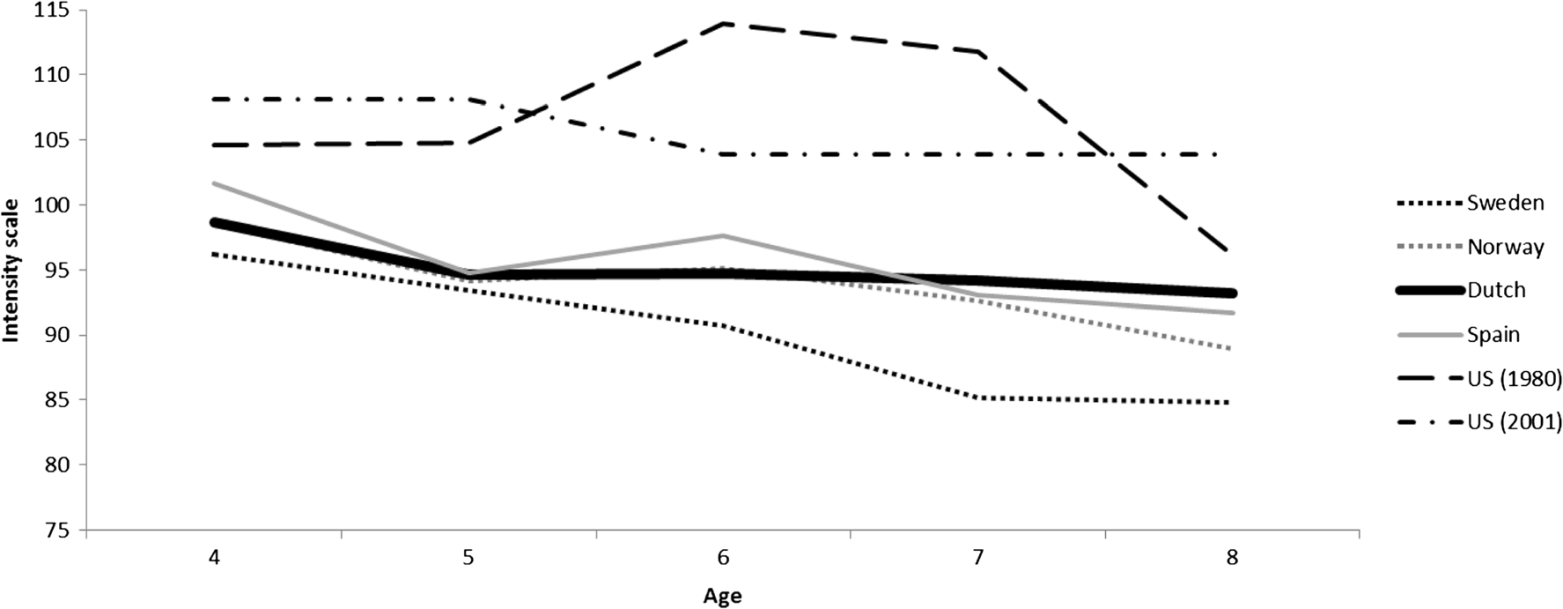
Intensity scale mean scores per country. *Note.* US 2001 study is a mean score of the used age groups 2–5 and 6–9 years

**Fig. 2:**
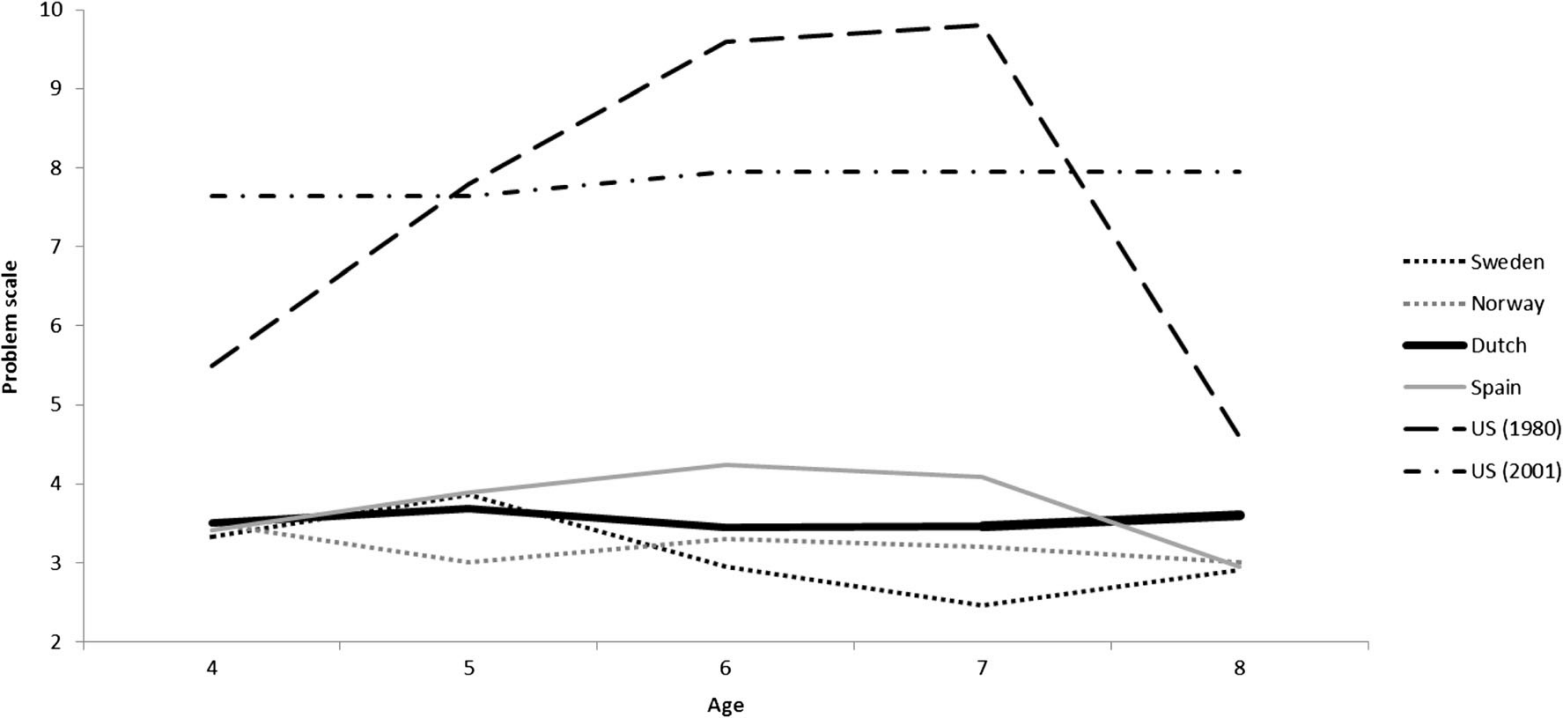
Problem scale mean scores per country. *Note.* US 2001 study is a mean score of the used age groups 2–5 and 6–9 years

Norm scores are available for the US (either age-specific, Robinson et al. 1980, or gender-specific, Burns and Patterson 2001), Norway (age- and gender-specific, Reedtz et al. 2008), Sweden (age- and gender-specific, Axberg et al. 2008), and Spain (age-specific, Calzada et al. 1998). A visual overview of the age-specific norm scores for these countries, including the Dutch age-specific norm scores is provided in Fig. 1 (Intensity scale) and Fig. 2 (Problem scale). Using independent samples t-test −using the mean, standard deviation, and sample size − we tested whether the Dutch mean score differed significantly from the mean scores reported in other countries. This procedure was similar to the one used by Axberg et al. (2008).

#### Comparison to US Norms

US parents rated their children’s disruptive behavior as more intense and more problematic than Dutch parents. Using independent samples t-test, comparing the original norm scores of the American children aged 4 to 8 years (*n* = 243; Robinson et al. 1980) to the Dutch scores, showed significant differences on both the intensity (*t*_intensity_ = 5.92, *p* < .001) and problem scale (*t*_problem_ = 10.77, *p* < .001). This also holds for the more recent US norm scores; comparing the US group of 6–9 year old children (*n* = 384; Burns and Patterson 2001) to the Dutch group of 6, 7 and 8 year old children (*n* = 3686) showed significant differences on both the Intensity (*t*_intensity_ = 8.69, *p* < .001) and Problem scale (*t*_problem_ = 18.72, *p* < .001).

#### Comparison to Scandinavian Norms

Comparing the scores of the Norwegian children (Reedtz et al. 2008) aged 4 to 8 (*n* = 2290) to the scores of the Dutch children, we found significant differences on both the Intensity (*t*_intensity_ = 5.18, *p* < .001) and Problem scale (*t*_problem_ = 3.35, *p* < .001). Norwegian parents rated their children’s disruptive behavior as less intense, as well as less problematic than Dutch parents. Swedish parents rated their children’s disruptive behavior as less intense but not less problematic than Dutch parents. Specifically, the scores of the Swedish 4 to 8 year old children (Axberg et al. 2008), were significantly lower on the Intensity scale (*t*_intensity_ = 5.05, *p* < .001), but not significantly lower on the Problem scale (*t*_problem_ = 1.81, *p* = .070).

#### Comparison to Spanish Norms

Spanish parents rated their children’s disruptive behavior similarly to Dutch parents, both on how frequent the behavior occurs and how problematic the behavior is. Comparing the scores of the Spanish children (Calzada et al. 1998) aged 4 to 8 (*n* = 265) to our group of 4–8 year old Dutch children showed no differences on the Intensity scale (*t*_intensity_ = 0.04, *p* = .966) or the Problem scale (*t*_problem_ = 0.53, *p* = .599).

## Discussion

The Eyberg Child Behavior Inventory (ECBI: Eyberg and Pincus 1999) is one of the most widely used and well-validated parent rating scales on child disruptive behavior. The scale has been successfully used for research and clinical purposes, in several countries including The Netherlands. To date, Dutch studies have relied on Scandinavian (Axberg et al. 2008; Reedtz et al. 2008) or US norm scores (Burns and Patterson 2001; Robinson et al. 1980). However, this may be problematic because of cross-cultural differences in the degree to which certain behaviors are seen as problematic by parents (Berg et al. 1997; Bilenberg 1999). The main goal of this paper therefore was to obtain Dutch norm scores among 6462 Dutch children aged 4 to 8 years (*M*_age_ = 6.37 years; *SD* = 1.32; 50.6% boys).

In line with previous research (Burns and Patterson 2001; Lahey et al. 2000; Lee et al. 2007) small but significant differences occurred on the mean sumscores across children of different ages (intensity scale) and gender (intensity and problem scale). In general, all children (boys as well as girls) showed a decrease in disruptive behavior when they grew older. However, boys generally showed disruptive behavior more frequently and parents perceived this behavior as more of a problem than for girls. It might indicate that boys simply show more disruptive behavior than girls do. This might be due to different gender norms and expectations and/or to biological differences. For example, distressed boys might act out, whereas girls might internalize more (e.g., Leadbeater et al. 1999). Moreover, when children get older they might learn to regulate their emotions and behavior better, which might be accompanied with a decrease in disruptive behavior over time (e.g., Miner and Clarke-Stewart 2008). Age and gender differences in disruptive behavior have been shown to be universal (Crijnen et al. 1997), and underline the importance of age- and gender-specific norms. Interestingly, our results showed that specifically disruptive behavior of children in the most rural areas was rated as less frequently occurring and were less perceived as a problem by their parents than the disruptive behavior of children in urban and less rural areas. This is in line with previous findings that disruptive behavior is more common in urban than rural areas, possibly due to higher social control in more rural areas (Elgar et al. 2003; Farrell et al. 2005; Hope and Bierman 1998).

We found significant differences between our norm scores and those of the US and Sweden and Norway. The Dutch norm scores on both the intensity and problem scale were lower than the US norm scores, but on the intensity scale (not the problem scale) higher than the Norwegian and Swedish norm scores. The differences between Dutch scores and US norms were in line with previous studies and our expectations. However, we did not necessarily expect the Dutch norm scores to be higher than the Scandinavian norms. There are different possible explanations for these cross-country differences in ECBI norm scores. On the one hand, it might be that the differences are explained by cross-cultural differences in parenting practices, and/or that disruptive behavior is simply more prevalent in some countries than in others. On the other hand, the explanation might lie in cultural values and perceptual differences. In some countries parents might perceive certain child behavior more easily as being disruptive than in other countries (e.g., Crijnen et al. 1997; Weisz et al. 1993). Indeed, studies have shown cultural differences in parents’ socialization goals, as well as the long term effects of certain parenting techniques on child behavior (e.g., Harwood et al. 1996; Lansford et al. 2005). Whatever the underlying explanation is, the differences in disruptive behavior scores between countries strongly underline the importance of identifying country-specific norm scores for children’s disruptive behavior. It shows that we cannot assume that the same clinical cut-off point is appropriate for children living in different (western) countries.

Our norm scores were also significantly higher than in a previous Dutch study on the ECBI (*n* = 177; *M* = 84.3; *SD* = 23.5; *t* = 4.78, *p* < .001; Abrahamse et al. 2015). The differences found between our study and that of Abrahamse and colleagues might be explained by different sample sizes and recruiting tactics. In the study by Abrahamse et al. (2015) participants were recruited at child day care centers, primary schools and through social networks without incentives. This recruiting tactic might have resulted in relatively high response rates in more advantaged families (i.e., high SES) whose children might generally show less problem behavior (Wadsworth and Achenbach 2005).

Our study has several limitations. First of all, although comparable to the 2001 US norm-study with a similar approach, response rate in this study was modest. Also, we did not ask parents for information on any demographics, such as gender, family composition or socio-economic background. These limitations are related; there are several reasons to suppose that the response rate might have been even lower without this anonymity, asking for more (personal) information. On the downside, this makes it more difficult to compare our families to the Dutch average and check whether our sample is indeed representative for all Dutch children. However, to include a heterogeneous sample we deliberately targeted areas that differed based on urbanity, mean income, and immigrant population. Based on the postal codes of participating families we found small differences between our sample population and the area population, but these differences were in both directions (i.e., our sample is scoring both below and above the area as well as the national mean). This suggests that there are no systematic differences between our sample and the area means. For example, we do not seem to have a systematic oversampling of families of high SES or families from Dutch origin. Also, we chose a very structured recruitment approach: all families with children in the targeted age group within the selected, both rural and urban, municipalities were approached. Moreover, offering a small incentive for returning the questionnaire is likely to have resulted in a heterogeneous group of responders (motivated to participate by different things). Indeed, based on the postal codes of our sample, the statistics on mean income and immigrant population show that there is large diversity in our sample and that the found differences between the targeted population and our sample were mostly small.

Second, we only asked one parent to fill out the ECBI. Because parents filled out the questionnaire without filling out any personal information, we do not know the gender of the parents who participated. High correlations are often found for father and mother reports on child disruptive behavior. For example, on the CBCL externalizing behavior scale agreement between mothers and fathers was 82.8% (Grietens et al. 2004). However, although there were no differences between Dutch mothers and Dutch fathers on the ECBI problem scale, mothers scored higher on the intensity scale (i.e., reported more frequent disruptive behavior than fathers, Abrahamse et al. 2015). Future research should take into account that different informants (including mothers and fathers) might provide different and unique information on the development of child disruptive behavior (De Los Reyes and Kazdin 2005).

Also, the age-range of children for who the ECBI can be used is 2–18 years. The current study focused on young schoolchildren aged 4–8 years because we feel the behaviors addressed in the questionnaire are specifically relevant for assessment of disruptive behavior in this age range. However, norm-scores for the other age-groups are necessary for the use of ECBI across developmental periods. Finally, we did not measure possible social desirability in the way parents filled out the ECBI. Although early American research showed that the ECBI scores do not relate to scores on a social desirability scale (Robinson and Anderson 1983), later research did find that social desirability predicted parents’ score on the ECBI intensity scale (Brestan et al. 2003). Future research should therefore consider adding a social desirability scale, specifically in the Dutch context (e.g., the Marlowe-Crown Social Desirability Scale, Crowne and Marlowe 1960).

Despite these limitations our study contributes to the field in important ways. The ECBI is a commonly used instrument. Computing norm scores for children in The Netherlands is important for both clinical and empirical purposes. From a clinical perspective, norm scores are needed to identify which children are at risk. These children can only be identified when the child’s individual score is compared with the average of his or her specific peer group. Specifically, the age group between 4 and 8 years might be a critical period for screening for and intervening in this behavior because the onset of persistent disruptive behavior lies in this developmental period (Eron and Huesmann 1990; Newman et al. 1997; Prior et al. 2001). From an empirical perspective, norm scores enable the selection and description of a study-sample. Baseline disruptive behavior is only informative when it can be compared to a specific norm. Our norm scores are based on a large, and true community sample: within the targeted municipalities, which were thoughtfully chosen, all families with children between 4 and 8 years of age were invited. Therefore, these norm scores enable both clinical and empirical practices to use the ECBI for screening purposes and to establishing the level of disruptive behavior of Dutch boys and girls relative to their peers.

There are a few notices that might have implications for the Dutch ECBI. First, the items on siblings were relatively often left open by parents and the item on bedwetting had a low item-total correlation, which has been reported in other samples as well (e.g., Abrahamse et al. 2015). Reconsideration of the item on bedwetting might otherwise increase reliability and validity of the scales. For items which might not be applicable for some families (e.g., items on siblings for children without siblings and on bedwetting for children who wear diapers), a ‘not-applicable’ answering option would reduce missing values and increase validity of the answers. Also, the amount of missings on the problem scale was relatively high. We handled this using state of the art full information maximum likelihood (FIML) estimations. However, a more detailed instruction for filling out that specific scale might prevent missingness.

In sum, the ECBI is a parent rating scale on disruptive behavior in children that is easy to administer, score, and interpret. Our study underlines the importance of identifying country-, age-, and gender-specific norms for disruptive behavior and provides ECBI norm scores for the Dutch population. Such norm scores improve the value of the instrument for empirical and clinical purposes, because it enables the use of the ECBI for screening Dutch children in terms of risk.

## References

[CR1] Abrahamse ME, Junger M, Leijten PHO, Lindeboom R, Boer F, Lindauer RJL (2015). Psychometric properties of the Dutch Eyberg child behavior inventory (ECBI) in a community sample and a multi-ethnic clinical sample. Journal of Psychopathology and Behavioral Assessment.

[CR2] Abrahamse ME, Junger M, van Wouwe MAMM, Boer F, Lindauer RJL (2016). Treating child disruptive behavior in high-risk families: A comparative effectiveness trial from a community-based implementation. Journal of Child and Family Studies.

[CR3] Achenbach, T.M., & Rescorla, L.A. (2001). Manual for the ASEBA School-age Forms & Profiles. Burlington: University of Vermont, Research Center for Children, Youth, & Families.

[CR4] Axberg U, Johansson Hanse J, Broberg AG (2008). Parents’ description of conduct problems in their children - a test of the Eyberg child behavior inventory (ECBI) in a Swedish sample aged 3-10. Scandinavian Journal of Psychology.

[CR5] Berg I, Fombonne E, McGuire R, Verhulst F (1997). A cross cultural comparison of French and Dutch disturbed children using the child behaviour checklist (CBCL). European Child & Adolescent Psychiatry.

[CR6] Bilenberg, N. (1999). The child behavior checklist (CBCL) and related material: Standardization and validation in Danish population based and clinically based samples. Acta Psychiatrica Scandinavica, *100*(S398), 2–52. doi:10.1111/j.1600-0447.1999.tb10703.x. 10.1111/j.1600-0447.1999.tb10703.x 10687023

[CR7] Boggs SR, Eyberg S, Reynolds LA (2010). Concurrent validity of the Eyberg child behavior inventory. Journal of Clinical Child Psychology.

[CR8] Brestan EV, Eyberg SM, Algina J, Johnson SB, Boggs SR (2003). How annoying is it? Defining parental tolerance for child misbehavior. Child & Family Behavior Therapy.

[CR9] Burns GL, Patterson DR (2001). Normative data on the Eyberg child behavior inventory and Sutter-Eyberg student behavior inventory: Parent and teacher rating scales of disruptive behavior problems in children and adolescents. Child & Family Behavior Therapy.

[CR10] Burns GL, Patterson DR (2010). Factor structure of the Eyberg child behavior inventory: A parent rating scale of oppositional defiant behavior toward adults, inattentive behavior, and conduct problem behavior. Journal of Clinical Child Psychology.

[CR11] Butler AM (2013). Cross-racial measurement equivalence of the Eyberg child behavior inventory factors among low-income young African American and non-Latino white children. Assessment.

[CR12] Calzada, E. J., Eyberg, S. M., García-Tornel Florensa, S., Mas Alguacil, J. C., Vilamala Sera, C., Villena Collado, H., … Trinxant Doménech, A. (1998). Inventario Eyberg del comportamiento en niños. Normalización de la versión española y su utilidad para el pediatra extrahospitalario. *Anales Españoles de Pediatría: Publicación Oficial de La Asociación Española de Pediatría ( AEP ), ISSN 0302–4342, Vol. 48, N*^*o*^*. 5 (MAYO), 1998, Págs. 475–482*, *48*(5), 475–482. 9656533

[CR13] Centraal Bureau voor de Statistiek (CBS). (2013). *Kerncijfers wijken en buurten*, 2013 Retrieved from https://www.cbs.nl/nl-nl/maatwerk/2015/36/kerncijfers-wijken-en-buurten-2013.

[CR14] Chhangur RR, Weeland J, Overbeek G, Matthys W, de Castro BO (2012). ORCHIDS: An observational randomized controlled trial on childhood differential susceptibility. BMC Public Health.

[CR15] Cohen J (1969). Statistical power analysis for the behavioural sciences.

[CR16] Crijnen AAM, Achenbach TM, Verhulst FC (1997). Comparisons of problems reported by parents of children in 12 cultures: Total problems, externalizing, and internalizing. Journal of the American Academy of Child & Adolescent Psychiatry.

[CR17] Crowne DP, Marlowe D (1960). A new scale of social desirability independent of psychopathology. Journal of Consulting Psychology.

[CR18] De Los Reyes A, Kazdin AE (2005). Informant discrepancies in the assessment of childhood psychopathology: A critical review, theoretical framework, and recommendations for further study. Psychological Bulletin.

[CR19] Elgar FJ, Arlett C, Groves R (2003). Stress, coping, and behavioural problems among rural and urban adolescents. Journal of Adolescence.

[CR20] Eron, L. D., & Huesmann, L. R. (1990). The stability of aggressive behavior—Even unto the third generation. In M. Lewis, S.M. Miller (eds), *Handbook of developmental psychopathology* (pp. 147–156). Boston: Springer US. 10.1007/978-1-4615-7142-1_12.

[CR21] Eyberg, S. M., & Pincus, D. (1999). *Eyberg child behavior inventory and sutter-eyberg student behavior inventory-revised: Professional manual*. Odessa: Psychological Assessment Resources.

[CR22] Farrell AD, Sullivan TN, Esposito LE, Meyer AL, Valois RF (2005). A latent growth curve analysis of the structure of aggression, drug use, and delinquent behaviors and their interrelations over time in urban and rural adolescents. Journal of Research on Adolescence.

[CR23] Goodman R (2001). Psychometric properties of the strengths and difficulties questionnaire. Journal of the American Academy of Child & Adolescent Psychiatry.

[CR24] Grietens H, Onghena P, Prinzie P, Gadeyne E, Van Assche V, Ghesquière P, Hellinckx W (2004). Comparison of mothers', fathers', and teachers' reports on problem behavior in 5-to 6-year-old children. Journal of Psychopathology and Behavioral aAsessment.

[CR25] Gross D, Fogg L, Young M, Ridge A, Cowell J, Sivan A, Richardson R (2007). Reliability and validity of the Eyberg child behavior inventory with African–American and Latino parents of young children. Research in Nursing & Health.

[CR26] Harwood RL, Schoelmerich A, Ventura-Cook E, Schulze PA, Wilson SP (1996). Culture and class influences on Anglo and Puerto Rican Mothers' beliefs regarding long-term socialization goals and child behavior. Child Development.

[CR27] Hope TL, Bierman KL (1998). Patterns of home and school behavior problems in rural and urban settings. Journal of School Psychology.

[CR28] Jokela M, Ferrie J, Kivimäki M (2009). Childhood problem behaviors and death by midlife: The British National Child Development Study. Journal of the American Academy of Child & Adolescent Psychiatry.

[CR29] Kingston L, Prior M (1995). The development of patterns of stable, transient, and school-age onset aggressive behavior in young children. Journal of the American Academy of Child & Adolescent Psychiatry.

[CR30] Lahey BB, Schwab-Stone M, Goodman SH, Waldman ID, Canino G, Rathouz PJ (2000). Age and gender differences in oppositional behavior and conduct problems: A cross-sectional household study of middle childhood and adolescence. Journal of Abnormal Psychology.

[CR31] Lansford JE, Chang L, Dodge KA, Malone PS, Oburu P, Palmérus K (2005). Physical discipline and children's adjustment: Cultural normativeness as a moderator. Child Development.

[CR32] Leadbeater BJ, Kuperminc GP, Blatt SJ, Hertzog C (1999). A multivariate model of gender differences in adolescents' internalizing and externalizing problems. Developmental Psychology.

[CR33] Lee K-H, Baillargeon RH, Vermunt JK, Wu H-X, Tremblay RE (2007). Age differences in the prevalence of physical aggression among 5–11-year-old Canadian boys and girls. Aggressive Behavior.

[CR34] Leijten P, Raaijmakers MAJ, Orobio de Castro B, Van den Ban E, Matthys W (2017). Effectiveness of the incredible years parenting program for families with socioeconomically disadvantaged and ethnic minority backgrounds. Journal of Clinical Child & Adolescent Psychology.

[CR35] Maughan B, Rowe R, Messer J, Goodman R, Meltzer H (2004). Conduct disorder and oppositional defiant disorder in a national sample: Developmental epidemiology. Journal of Child Psychology and Psychiatry.

[CR36] Menting AT, Orobio de Castro B, Wijngaards-de Meij LD, Matthys W (2014). A trial of parent training for mothers being released from incarceration and their children. Journal of Clinical Child & Adolescent Psychology.

[CR37] Miner JL, Clarke-Stewart KA (2008). Trajectories of externalizing behavior from age 2 to age 9: Relations with gender, temperament, ethnicity, parenting, and rater. Developmental Psychology.

[CR38] Newman DL, Caspi A, Moffitt TE, Silva PA (1997). Antecedents of adult interpersonal functioning: Effects of individual differences in age 3 temperament. Developmental Psychology.

[CR39] Posthumus JA, Raaijmakers MAJ, Maassen GH, van Engeland H, Matthys W (2012). Sustained effects of incredible years as a preventive intervention in preschool children with conduct problems. Journal of Abnormal Child Psychology.

[CR40] Prior M, Smart D, Sanson A, Oberklaid F (2001). Longitudinal predictors of behavioural adjustment in pre-adolescent children. Australian and New Zealand Journal of Psychiatry.

[CR41] Reedtz C, Bertelsen B, Lurie J, Handegård BH, Clifford G, Morch WT (2008). Eyberg child behavior inventory (ECBI): Norwegian norms to identify conduct problems in children: Development and aging. Scandinavian Journal of Psychology.

[CR42] Rich BA, Eyberg SM (2001). Accuracy of assessment: The discriminative and predictive power of the Eyberg child behavior inventory. Ambulatory Child Health.

[CR43] Robinson EA, Anderson LL (1983). Family adjustment, parental attitudes, and social desirability. Journal of Abnormal Child Psychology.

[CR44] Robinson, E. A., Eyberg, S. M., & Ross, A. W. (1980). The standardization of an inventory of child conduct problem behaviors. *Journal of Clinical Child Psychology, 9*, 22–28. 10.1080/15374418009532938.

[CR45] Spijkers W, Jansen DE, Reijneveld SA, Theunissen M, Vogels A, Reijneveld S (2013). Effectiveness of primary care triple P on child psychosocial problems in preventive child healthcare: A randomized controlled trial. BMC Medicine.

[CR46] Timimi S, Taylor E (2004). ADHD is best understood as a cultural construct. The British Journal of Psychiatry.

[CR47] Villodas MT, Litrownik AJ, Thompson R, Jones D, Roesch SC, Hussey JM (2015). Developmental transitions in presentations of externalizing problems among boys and girls at risk for child maltreatment. Development and Psychopathology.

[CR48] Von Stumm S, Deary IJ, Kivimäki M, Jokela M, Clark H, Batty GD (2011). Childhood behavior problems and health at midlife: 35-year follow-up of a Scottish birthcohort. Journal of Child Psychology and Psychiatry.

[CR49] Wadsworth ME, Achenbach TM (2005). Explaining the link between low socioeconomic status and psychopathology: Testing two mechanisms of the social causation hypothesis. Journal of Consulting and Clinical Psychology.

[CR50] Weeland J, Chhangur RR, van der Giessen D, Matthys W, Orobio de Castro B, Overbeek G (2017). Intervention effectiveness of the incredible years: New insights into sociodemographic and intervention-based moderators. Behavior Therapy.

[CR51] Weis R, Lovejoy MC, Lundahl BW (2005). Factor structure and discriminative validity of the Eyberg child behavior inventory with young children. Journal of Psychopathology and Behavioral Assessment.

[CR52] Weisz JR, Sigman M, Weiss B, Mosk J (1993). Parent reports of behavioral and emotional problems among children in Kenya, Thailand, and the United States. Child Development.

[CR53] Wothke, W. (2000). Longitudinal and multigroup modeling with missing data. In Little, Todd D. (Ed); Schnabel, Kai U. (Ed); Baumert, Jürgen (Ed). (2000). Modeling longitudinal and multilevel data: Practical issues, applied approaches, and specific examples, (pp. 219-240, 269-281). Mahwah: Lawrence Erlbaum Associates Publishers.

